# Lung ultrasound: The future ahead and the lessons learned from COVID-19a)

**DOI:** 10.1121/10.0002183

**Published:** 2020-10-16

**Authors:** Libertario Demi

**Affiliations:** Ultrasound Laboratory Trento (ULTRa), Department of Information Engineering and Computer Science, University of Trento, Via Sommarive 9, 38123, Trento, Italy

## Abstract

Lung ultrasound (LUS) is a rapidly evolving field of application for ultrasound technologies. Especially during the current pandemic, many clinicians around the world have employed LUS to assess the condition of the lung for patients suspected and/or affected by COVID-19. However, LUS is currently performed with standard ultrasound imaging, which is not designed to cope with the high air content present in lung tissues. Nowadays LUS lacks standardization and suffers from the absence of quantitative approaches. To elevate LUS to the level of other ultrasound imaging applications, several aspects deserve attention from the technical and clinical world. This overview piece tries to provide the reader with a forward-looking view on the future for LUS.

Here we are, four months after the World Health Organization declared on March 11, 2020 that coronavirus diseases 2019 (COVID-19) could be characterized as a pandemic. The first cases were detected in Wuhan, a city located in Hubei province, China, in December 2019.^1^ At the end of January, 7818 cases were confirmed globally. Of these, 82 cases were reported in 19 countries outside of China, namely Japan (11), Republic of Korea (4), Vietnam (2), Singapore (10), Australia (7), Malaysia (7), Cambodia (1), Philippines (1), Thailand (14), Nepal (1), Sri Lanka (1), India (1), United States of America (5), Canada (3), France (5), Finland (1), Germany (4), and United Arad Emirates (4).^2^ By the end of February, the number of confirmed cases grew to 85 403, with 54 countries reporting confirmed cases.^3^ On March 31, 2020, 750 890 cases and 36 405 deaths were confirmed globally. Western countries were strongly hit. In Europe, Italy, Spain, Germany, and France reported 101 739, 85 195, 61 913, and 43 977 cases, respectively. The United States of America reported 140 640 cases. In comparison, the total confirmed cases in China were 82 545.^4^

As of July 12, 2020, a total of 12 552 765 confirmed cases and 561 617 deaths were reported worldwide. The Americas were seeing the highest number of cases, with the United States of America and Brazil reporting 3 163 581 and 1 800 827 cases, respectively.^5^

COVID-19 is caused by severe acute respiratory syndrome coronavirus 2 (SARS-CoV-2), a virus that causes serious complications to the respiratory system. Symptomatology differs significantly from patient to patient. The most common symptoms include fever, dry cough, and tiredness. Other less common symptoms are aches and pains, nasal congestion, headache, conjunctivitis, sore throat, diarrhoea, loss of taste or smell, a rash on skin, and discoloration of fingers or toes. Generally, symptoms are mild, and the majority of the patients (around 80%) do not need hospital treatment. However, about one in five becomes seriously ill.^6^

The need of a timely and correct diagnosis, as well as the importance of patient monitoring, have clearly emerged from the very beginning.^1^ To this end, reverse transcription polymerase chain reaction testing has been widely adopted as a diagnostic tool.^7^ Essentially, this test is based on the detection of viral ribonucleic acid in respiratory secretions collected by nasopharyngeal swab. However, test availability has been a big concern, and a high false-negative rate has been reported.^8^

Chest computed tomography (CT) has also been used as a diagnostic tool, with reported sensitivity and specificity in the 61%–99% and 25%–33% range, respectively.^9^ However, chest CT is based on ionizing radiation, is not portable or generally available in every setting, and requires time-expensive disinfection procedures and moving patients within the hospital facilities, which increases the risk of contamination.

For all these reasons, lung ultrasound (LUS) has been receiving growing attention as an alternative imaging modality in the current pandemic context. In fact, thanks to its capability to provide information in real time and its portability, safety, and wide availability, LUS has been suggested to be a valuable tool in a wide variety of settings.^10,11^ For example, LUS can be applied for triage of symptomatic patients, both at home as well as in the pre-hospital phase. It could also serve for stratification and monitoring, on the basis of the extension of specific LUS patterns and their temporal evolution toward the consolidation phase. LUS safety is also particularly indicated for specific patient populations, such as pregnant women.^12,13^ Moreover, LUS could play a role in the definition and monitoring of treatment. Ultimately, LUS can significantly reduce the risk of exposure of health care personnel.^14^

However, although LUS has clearly proven its diagnostic value and capability to intercept alterations along the lung surface even before COVID-19, its current use is still mainly limited to the qualitative and subjective analysis of imaging artifacts.^15^ To give to the reader a clear example, one of the most important artifacts in LUS is represented by B-lines. A correlation exists between B-lines and the increase in extravascular lung water,^16^ interstitial lung diseases,^17,18^ cardiogenic and non-cardiogenic lung oedema,^19^ interstitial pneumonia,^20^ and lung contusion.^21^ B-lines are defined as “*discrete laser-like vertical hyperechoic reverberation artifacts that arise from the pleural line (previously described as ‘comet tails’), extend to the bottom of the screen without fading, and move synchronously with lung sliding*.”^22^ To an ultrasound expert, it is evident that such a definition is ambiguous. What does laser-like mean? Does this definition depend on the lateral extent of the artifact? What if the imaging depth is changed, and the artifact no longer reaches the bottom of the screen? Is there a minimum length below which a vertical artifact cannot be called a B-line? What are the guidelines with respect to the gain and time-gain-compensation settings? What does without fading mean exactly, and is there a specific dynamic range to consider?

These and other key questions clearly need to be addressed if we are to develop a reproducible, accurate, and reliable ultrasound method dedicated to the lung. Moreover, standardization is essential, especially when considering key aspects such as probe selection and imaging setting optimization. This remains the biggest limitation for LUS today, complicating reproducibility and accuracy.

In the context of COVID-19, progress in this direction has been made. A proposal for standardization of the use of LUS was recently presented.^23^ A 4 level scoring system is proposed, together with a detailed imaging protocol that defines imaging landmarks, acquisition time, probes, and imaging settings, such as focal point location and mechanical index. The 4 levels are defined based on the current understanding of ultrasound interaction with lung tissue. Score 0 is linked to a continuous pleural line and associated horizontal artifacts. These are generally referred to as A-lines, and are due to the high reflectivity of the non-pathological lung surface hindering ultrasound propagation beyond the pleural line. Consequently, ultrasound waves scatter multiple times between the lung surface and the probe, giving rise to this specific pattern. Score 1 is associated with the appearance of the first abnormalities. Specifically, the pleural line is not continuous anymore and vertical artifacts are visible. Here, the general term vertical artifact was used to avoid the ambiguity related to the definition of B-lines. Moreover, the presence, and not the number, of vertical artifacts is evaluated. In fact, recent *in vitro* and clinical studies showed how the imaging frequency and bandwidth strongly influence the visualization of these patterns.^24,25^ Vertical artifacts are not specific to COVID-19, and simply signal the presence of local alterations along the lung surface. Their visualization during an ultrasound examination is considered to be linked to the formation, along the lung surface, of channels accessible to ultrasound, which can be generated in many pathological states of the lung once volumes originally filled by air are occupied by media that are acoustically much more similar to the intercostal tissue (e.g., water, blood, and tissue).^25^

Score 2 is associated with the appearance of small-to-large consolidated areas. These are not artifacts, but anatomical findings that appear as hypoechoic areas along the lung surface. The loss of echogenicity of the consolidated areas is linked to the loss of aeration and to the transition of these areas toward acoustic properties similar to soft tissue. Below the consolidations, vertical artifacts are generally found. The latter are most likely associated with the presence of areas not yet fully deaerated. Ultimately, a score 3 is associated with the presence of large, extended (>50% of the pleural line) vertical artifacts, with or without large consolidations. Figure 1 shows examples of the above described patterns as visualized with both linear and convex probes. This scoring system has already been tested within a preliminary clinical study, where the distribution of the different scores with respect to the 14 landmarks was investigated and compared with chest CT findings.^26^ This imaging protocol represents a trade-off between an extended evaluation of the lung surface and the duration of the examination. A higher number of landmarks would, in fact, guarantee a richer data set on which to base the analysis, but requires lengthier examinations. On the other hand, reducing the number of landmarks increases the chances of missing important findings. This is especially relevant for patients affected by COVID-19.^26^

**FIG. 1.**
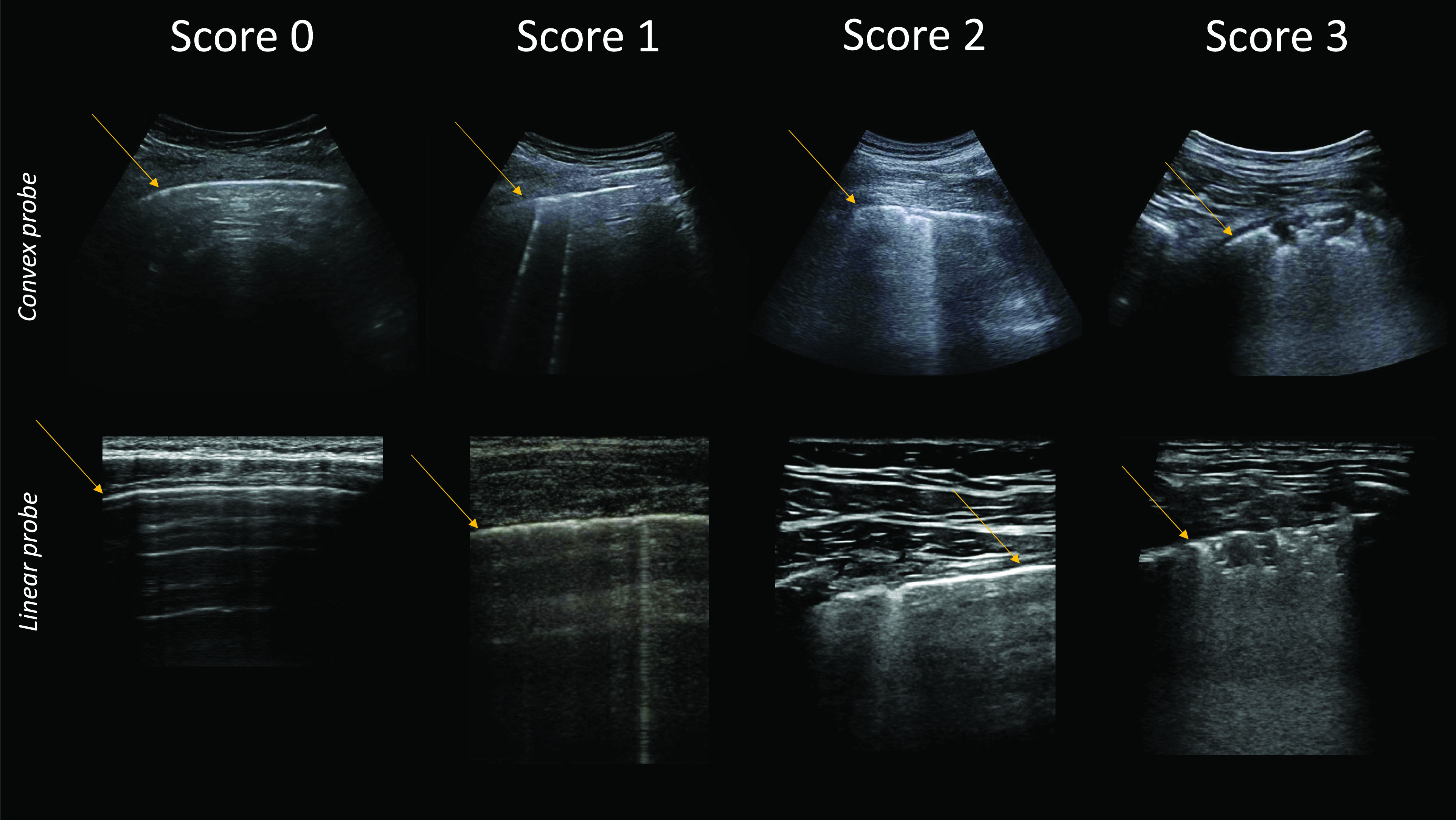
(Color online) LUS patterns that characterize the different score levels, as visualized when employing a convex (top) or linear (bottom) probe. Orange arrows indicate the location of the pleural line.

This type of study is important for the definition of the optimal image acquisition protocol. In fact, it is fundamental to define how many LUS videos should be acquired and where, to correctly evaluate the status of the lung of a patient. While keeping the number of acquisitions low is preferable to reduce scanning time, an excessively reduced amount could lead to significant underestimations. The study included 38 patients. The most severe findings were found in the posterior regions. Specifically, 21.05% of posterior areas were classified as score 3. In comparison, only 13.62% of lateral areas and 5.92% of anterior areas were classified as score 3. Chest CT findings correlated and matched the distribution of LUS findings. Recently, other studies are also comparing LUS with CT findings.^27,28^

Beyond the standardization of the acquisition protocol and scoring system, dedicated algorithms have been developed for the automatic evaluation of LUS videos.^29,30^ These algorithms provide frame and video based scoring as well as segmentation of the patterns of interest in a few seconds, and are now freely available through a web-application (https://iclus-web.bluetensor.ai/). In this way, clinicians can obtain a reproducible evaluation of their LUS data.

Extended clinical studies aimed at defining the prognostic value of the above described scoring system are currently ongoing.

Compared to what is described above, other studies focused attention on subjective definitions of imaging artifacts, ending up proposing different names and qualitative descriptions for the same pattern.^31,32^

Ultimately, another interesting area of research is now emerging where the use of contrast-enhanced ultrasound (CEUS) imaging for characterizing consolidations is investigated.^33,34^ Particularly, the understanding of the perfusion level of the consolidated areas could be highly significant for the patho-genetic interpretation of the consolidations in COVID-19 and, consequently, for the definition of the optimal treatment.

Moreover, there is certainly room for the development of computer aided systems, model and artificial intelligence (AI) based, which can help the clinicians not only in the evaluation of the videos but also guide them accordingly to predefined protocols during the acquisition process with real time feedbacks. This would be of tremendous support for the generation of standardized data sets, which are essential for further developments in LUS, and speed up clinicians' learning curve. In other contexts, AI systems have already been applied to guide ultrasound imaging.^35^ Once extensively proven and validated, these technologies may ultimately enable non-experts to perform LUS investigations.

To conclude, multiple lessons can be learned from the current pandemic. First, LUS has proved again to be a valuable support tool in the diagnosis of lung diseases. This is particularly true in a scenario in which patients' mobility and health care professionals' exposure should be minimized, and diagnostic instrumentations should be widely accessible and not concentrated in a few facilities. Second, clinicians and technical experts should cooperate side by side aiming at standardizing image acquisition protocols and image analysis tools. Failing to do so will harm the credibility of LUS and hinder the exploitation of its full potential. Third, specific studies aimed at investigating LUS safety limits are needed. In fact, studies on animal lung tissue have reported effects such as pulmonary capillary haemorrhage for mechanical indices in the diagnostic range.^36^ Fourth, much remains to be understood with respect to the interaction between ultrasound waves and the lung. Specifically, studies aimed at understanding the genesis of LUS artifacts are needed. Moreover, characterising the ultrasound signals backscattered by a pathological lung and defining specific correlations between this characterization and the underlying pathology are crucial if we are to improve LUS specificity. To conclude, the future of LUS lies in the development of ultrasound methods designed around the specific properties of lung tissue and ultimately guarantee a truly quantitative analysis. Efforts in this direction are currently emerging, with interesting results already obtained from controlled experiments on lung-mimicking phantoms^24,37^ and animal models^38^ and from the first clinical studies.^39,40^
